# Cardiac energetics in severe mitral regurgitation: relationship with eccentric hypertrophy, stroke volume, and effects of valve repair

**DOI:** 10.1093/ehjimp/qyaf146

**Published:** 2025-11-25

**Authors:** Mark A Peterzan, William T Clarke, Hannah A Lake, David Dearlove, John A Henry, Andrew J M Lewis, Moritz J Hundertmark, Jennifer J Rayner, Andrew P Apps, William D Watson, Rana A Sayeed, Craig A Lygate, Stefan Neubauer, Christopher T Rodgers, Oliver J Rider

**Affiliations:** Radcliffe Department of Medicine, Division of Cardiovascular Medicine, University of Oxford Centre for Clinical Magnetic Resonance Research, University of Oxford, John Radcliffe Hospital, Oxford OX3 9DU, UK; Wellcome Centre for Integrative Neuroimaging, FMRIB, Nuffield Department of Clinical Neurosciences, University of Oxford, Oxford, UK; Division of Cardiovascular Medicine, Radcliffe Department of Medicine, University of Oxford, Oxford, UK; Department of Physiology, Anatomy and Genetics, University of Oxford, Oxford, UK; Radcliffe Department of Medicine, Division of Cardiovascular Medicine, University of Oxford Centre for Clinical Magnetic Resonance Research, University of Oxford, John Radcliffe Hospital, Oxford OX3 9DU, UK; Radcliffe Department of Medicine, Division of Cardiovascular Medicine, University of Oxford Centre for Clinical Magnetic Resonance Research, University of Oxford, John Radcliffe Hospital, Oxford OX3 9DU, UK; Radcliffe Department of Medicine, Division of Cardiovascular Medicine, University of Oxford Centre for Clinical Magnetic Resonance Research, University of Oxford, John Radcliffe Hospital, Oxford OX3 9DU, UK; Radcliffe Department of Medicine, Division of Cardiovascular Medicine, University of Oxford Centre for Clinical Magnetic Resonance Research, University of Oxford, John Radcliffe Hospital, Oxford OX3 9DU, UK; Radcliffe Department of Medicine, Division of Cardiovascular Medicine, University of Oxford Centre for Clinical Magnetic Resonance Research, University of Oxford, John Radcliffe Hospital, Oxford OX3 9DU, UK; Radcliffe Department of Medicine, Division of Cardiovascular Medicine, University of Oxford Centre for Clinical Magnetic Resonance Research, University of Oxford, John Radcliffe Hospital, Oxford OX3 9DU, UK; Department of Cardiothoracic Surgery, Oxford Heart Centre, John Radcliffe Hospital, Oxford, UK; Division of Cardiovascular Medicine, Radcliffe Department of Medicine, University of Oxford, Oxford, UK; Radcliffe Department of Medicine, Division of Cardiovascular Medicine, University of Oxford Centre for Clinical Magnetic Resonance Research, University of Oxford, John Radcliffe Hospital, Oxford OX3 9DU, UK; Department of Clinical Neurosciences, Wolfson Brain Imaging Centre, University of Cambridge, Cambridge, UK; Radcliffe Department of Medicine, Division of Cardiovascular Medicine, University of Oxford Centre for Clinical Magnetic Resonance Research, University of Oxford, John Radcliffe Hospital, Oxford OX3 9DU, UK

**Keywords:** mitral regurgitation, myocardial energetics, phosphorous spectroscopy, PCr/ATP, mitral valve repair

## Abstract

**Aims:**

Understanding changes in ATP metabolism may lead to improved risk stratification in severe primary mitral regurgitation (MR). Here, we seek to compare the energetic phenotype of volume-overload pathological hypertrophy with athletic hypertrophy and with the normal heart under catecholamine stress.

**Methods and results:**

Nineteen severe-MR patients underwent cardiac magnetic resonance and ^31^P-spectroscopy for energetics, including phosphocreatine to adenosine triphosphate ratio (PCr/ATP), the pseudo-first-order forward rate constant of the creatine kinase reaction (*k*_f_) and CK flux (*k*_f_ × [PCr]). When compared with 20 healthy controls, severe MR was associated with lower PCr/ATP (1.58 ± 0.32 vs. 2.08 ± 0.28, *P* < 0.001). This is related to the severity of regurgitation (*r* −0.59, *P* < 0.001) but not to LVEF (*r* −0.20, *P* = 0.23) or LV systolic strain (*P* = 0.18). When compared to 17 athletes with similarly increased end-diastolic volume (athletes 107 ± 10 mL/m^2^ vs. 114 ± 22, *P* = 0.29), severe MR had greater total cardiac output (by 42%, *P* < 0.001), and lower PCr/ATP (by 28%, *P* < 0.001) and CK flux (by 41%, *P* = 0.04). When compared to normal hearts during dobutamine stress at matched cardiac output levels, median *k*_f_ (by 45%, *P* = 0.08) and CK flux (by 53%, *P* = 0.02) were lower in severe MR. PCr/ATP increased (by 17%, *P* = 0.04) following mitral valve repair (MVR) in a subset of patients (*n* = 14, median 7 months). Seven patients during MVR and six patients without volume loading donated LV biopsy, revealing that creatine was not lower in severe MR.

**Conclusion:**

Even with normal LVEF, severe MR is associated with reduced PCr/ATP, CK *k*_f_, and CK flux. PCr/ATP reduction resolved with MVR. Thus, targeting CK capacity and/or flux may be a therapeutic strategy to prevent/treat systolic failure in MR.

## Graphical Abstract

**Graphical Abstract:**
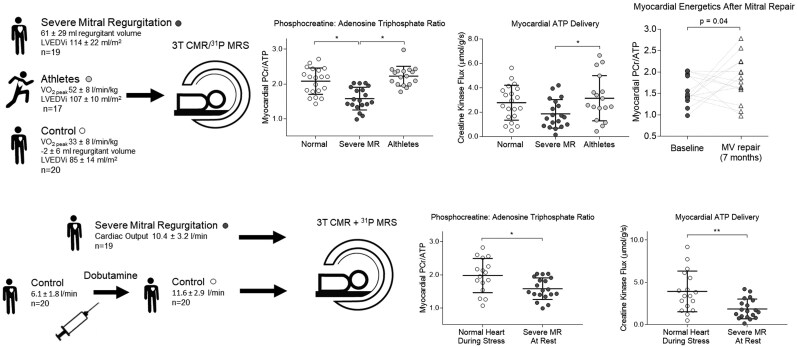

## Introduction

The timing of mitral surgery remains one of the most difficult problems of clinical cardiology. Current guidelines recommend repair for symptomatic severe valvular disease, or asymptomatic severe valvular disease with evidence of detrimental pathophysiological changes, such as left ventricular systolic dysfunction, pulmonary hypertension, or atrial fibrillation.^1^

However, symptoms can remain minimal despite severe regurgitation as a result of the adaptive eccentric remodelling of left ventricle (LV) and atrium, which allows increased cardiac output while reducing LV end diastolic pressure and preventing pulmonary congestion.^2^ In addition, the low-pressure run-off into the left atrium causes systolic unloading of the LV, which can flatter LV ejection fraction (LVEF) and other volumetric measures of systolic function.^3^

As a result, despite adherence to current guidelines^4^ a significant proportion of patients will develop early post-operative LVEF decline that is greater than expected from the reduction in MR alone.^5^ Global longitudinal strain has been proposed as a more sensitive measure,^6^ but is also affected by loading conditions, making it subject to the same problems as LVEF. As a result, newer markers of risk are needed that are independent from the effects of LV systolic unloading.

Given the direct coupling of adenosine triphosphate (ATP) utilization and LV contractile performance, changes in ATP metabolism have the potential to represent an early marker of susceptibility to LV contractile decompensation (transition to failure). In line with this, the phosphocreatine to adenosine triphosphate ratio (PCr/ATP) has previously been shown to be reduced in mitral regurgitation (MR).^7^ It is unknown, however, whether or not this energetic depletion is related to mitral regurgitant severity, the process of LV eccentric remodelling, or if it is independent from LVEF, or reversible with mitral valve repair (MVR). It has also recently been shown that ATP delivery to the myocardium can be maintained in the face of a reduced PCr/ATP via an increase in creatine kinase activity,^8^ but whether or not ‘compensatory’ increased CK activity exists in MR is also not known. As such, whilst attractive as a marker of risk in MR, further understanding of myocardial energetics in MR is needed.

## Methods

The work was prospectively approved by the regional ethics committee (South Central Oxford C, 16/SC/0323) with written consent prior to all procedures. Research was carried out in accordance with institutional procedures and the principles of the Declaration of Helsinki. The data underlying this article will be shared on reasonable request to the corresponding author.

### Subjects

Nineteen patients with severe primary MR, preserved systolic function on echocardiography, and a guideline-indicated decision for surgical MVR were recruited. Seventeen trained athletes [criteria; ≥ 8 h weekly endurance training for ≥1 year peak VO_2_ index of >45 mL/min/kg (men only), and an anaerobic threshold (AT) > 70% peak VO_2_ index] were recruited. Twenty healthy volunteers (exercising <3 h per week) and patients with non-hypertrophied, non-volume-loaded hearts with normal systolic function undergoing cardiac surgery, willing to have biopsies taken were also recruited.

A priori power calculations were performed. Assuming ANOVA analysis (alpha 0.05, beta 0.8), the sample size for each group was estimated from the expected standard deviation (SD) and the expected effect size. The values for our sample size are based on previous work. Sample size calculations were performed with GPower3*. Based on human pilot data, to identify a 20% difference in CK flux (mean 0.2, SD 0.05), a sample size of 15 subjects was required. Patients were recruited from routine clinical lists over a 1-year period until recruitment target was reached, whilst healthy controls and athletes were recruited from public advertisements.

### Exclusion criteria

Exclusion criteria included; myocardial infarction, more than mild bystander valve disease, GFR < 30 kg/m^2^, pregnancy. Participants listed for cardiac surgery had flow-limiting atheroma excluded by invasive angiography and prior myocardial infarction excluded by late gadolinium cardiovascular magnetic resonance.

### Cardiovascular magnetic resonance

All CMR was performed on a 3T MRI scanner (Tim Trio; Siemens, Erlangen, Germany) and unless stated was analysed using cvi42 (Circle Cardiovascular Imaging Inc., Alberta, Canada).^9^ Expanded methods are detailed in the supplementary data.

### Left ventricular imaging

Standard ECG-gated, breath-hold short-axis LV stacks were obtained. LV parameters and 3D strain measures were derived from manually contoured images using feature tracking within cvi42.

### Mitral regurgitation quantification

Mitral regurgitant volume was calculated as aortic stroke volume minus LV stroke volume (SV), and from ventricular SV difference. Mitral regurgitant fraction was calculated as mitral regurgitant volume/LVSV.

### Dobutamine infusion

Dobutamine was infused at incremental rates up to 40 μg/kg/min to achieve a target heart rate of 65% maximum (calculated as 220-age). Cine images were repeated at stress for LV function and to exclude regional wall motion abnormalities, as previously described.^10^

### Cardiac ^31^P-magnetic resonance spectroscopy

#### Phosphocreatine:adenosine triphosphate ratio

A ∼10 min non-gated 3D acquisition-weighted ultra-short echo time CSI sequence was run, as previously described.^9^ Spectral analyses were performed using ‘OXSA’, an open-source MATLAB implementation of the AMARES algorithm.^11^

#### Creatine kinase pseudo first order forward rate constant (CK *k*_f_)

Resting CK *k*_f_ was estimated using triple repetition time saturation transfer (TRiST) as previously described.^12^ Spectral analysis was performed using custom software.^11^ Two further sets of ^31^P spectra were acquired during dobutamine to calculate stress CK *k*_f_, as previously described.^13^

### Cardiopulmonary exercise testing

Athletes and healthy volunteers underwent bicycle cardiopulmonary exercise testing with breath-by-breath respiratory gas measurement and individualized protocols. Minute oxygen consumption (VO_2_), heart rate, and AT as a percentage of peak VO_2_ are presented. One author (DD), blinded to group assignment, read the AT as the first inflection point of the VCO_2_/VO_2_ slope.

### Left ventricular biopsies

Seven of the 19 patients undergoing MVR and six patients with non-volume-loaded hearts had surgical myocardial biopsies that were obtained from LV endocardium 10–20 min after cardiopulmonary bypass was established. Biopsy samples were frozen in liquid nitrogen within 20 s of excision and stored at −80°C until analysis. Indications for surgery in biopsied non-volume-loaded patients were mitral stenosis (*n* = 4), ascending aortic aneurysm (*n* = 1), and benign left atrial mass (*n* = 1).

### Enzyme activities, CK isozyme distribution and total creatine content

Frozen, powdered samples were analysed according to established protocols for CK total activity (averaged over three runs, normalized to Lowry protein (mg/mL), presented as IU/mg protein), and total creatine concentration, i.e. the sum of free creatine and phosphocreatine (nmol/mg protein) using high performance liquid chromatography (HPLC), as previously described.^14^

### Statistical analysis

Data were reported as means (with SD) for continuous variables passing Shapiro–Wilk testing for normality and medians [with interquartile range (IQR)] if not. One, two, or three asterisks in figures indicate significance at *P* < 0.05, 0.01, and 0.001, respectively. Continuous variables were compared with parametric tests if the Shapiro–Wilk test and Levene test of between-group homogeneity of variance were passed. Welch's correction was used for unequal sample sizes or if the assumption of homogeneity of variance was violated. Multiple group comparisons were performed using one-way ANOVA with Bonferroni correction. If normality assumptions were not met, non-parametric tests were used. *P* ≤ 0.05 was used as a threshold of significance. Analyses were performed using R version 3.5.2 (R Foundation for Statistical Computing, Vienna, Austria) and SPSS (v25, IBM, New York, USA).

## Results

### Demographics, anthropometric and exercise capacity

Whilst healthy volunteer and athlete group ages were not different (*P* = 0.26, *Table 1*), participants with severe MR were older (*P* < 0.001, for both analyses, *Table 1*) and had higher systolic blood pressure than athletes (*P* = 0.005). Prior smoking was more common in both healthy volunteers and participants with severe MR groups than in athletes. Participants scheduled for MVR had higher use of cardiac medications (*Table 1*) than other groups. As expected, the athlete group had significantly higher peak VO_2_ (52.2 ± 7.9 vs. 33.2 ± 8.4 mL/min/kg, *P* < 0.001) and AT relative to VO_2_ peak (79.4 ± 6.6 vs. 48.0 ± 10.4%, *P* < 0.001) than healthy volunteers.

**Table 1:** Participant demographics, medical, and drug history

	Severe MR (*n* = 19)	Healthy volunteer (*n* = 20)	Athlete (*n* = 17)
**Anthropometric**			
Age (years)	67 (10)	37 (28–68)*	36 (10)
BMI (kg/m^2^)	27 (5)	24 (3)	23 (2)
Body surface area (m^2^)	1.93 (0.26)	1.88 (0.23)	1.96 (0.25)
Heart rate (/min)	67 (61–74)	60 (53–65)*	46 (38–54)
Systolic BP (mmHg)	133 (19)	123 (18)	116 (10)
Diastolic BP (mmHg)	72 (7)	67 (11)	66 (8)
Mean arterial pressure (mmHg)	92(9)	86(11)*	82(8)
**Exercise capacity/symptoms**			
NYHA 1 (%)	3 (16)	N/A	N/A
NYHA 2 (%)	13 (68)	N/A	N/A
NYHA 3 (%)	3 (16)	N/A	N/A
6MWT (m)	533 (96)	606 (63)	—
Peak VO_2_ (L/min)	—	2.45 (0.74)^#^	4.05 (0.89)
Peak VO_2_ (mL/min/kg)	—	33.2 (8.4)^#^	52.2 (7.9)
AT (L/min)	—	1.18 (0.43)^#^	3.23 (0.82)
AT relative to VO_2_ peak (%)	—	48.0 (10.4)^#^	79.4 (6.6)
**Comorbidities**			
Diabetes mellitus (%)	0 (0)	0 (0)	0 (0)
Hypertension (%)	6 (32)	1 (5)*	0 (0)
Venous thromboembolism (%)	0 (0)	0 (0)	0 (0)
TIA/stroke (%)	1 (5)	0 (0)	0 (0)
Smoker (%)	0 (0)	0 (0)	0 (0)
Prior smoker (%)	8 (42)	9 (45)	0 (0)
Atrial fibrillation (%)	4 (21)	0 (0)	0 (0)
**Medications**			
Beta-blocker (%)	7 (37)	2 (10)*	0 (0)
ACE-I/ARB (%)	9 (47)	1 (5)*	0 (0)
MRA (%)	2 (11)	0 (0)	0 (0)
Loop diuretic (%)	8 (42)	0 (0)*	0 (0)
Antiplatelet (%)	3 (16)	0 (0)	0 (0)
OAC (%)	4 (21)	0 (0)	0 (0)

Counts given as number (%); continuous variables as mean (SD) or median (IQR). Parameters differing significantly between severe MR and healthy volunteers are marked * *P* < 0.05. Parameters differing significantly between athletes and healthy volunteers are marked ^#^  *P* < 0.05. NYHA, New York Heart Association symptom class; 6mwt, 6-min walk distance; TIA, transient ischaemic attack; ACE-I, angiotensin-converting enzyme inhibitor; ARB, angiotensin receptor blocker; MRA, mineralocorticoid receptor antagonist; OAC, oral anticoagulant.

### Cardiac volumes and geometry

**Figure 1:**
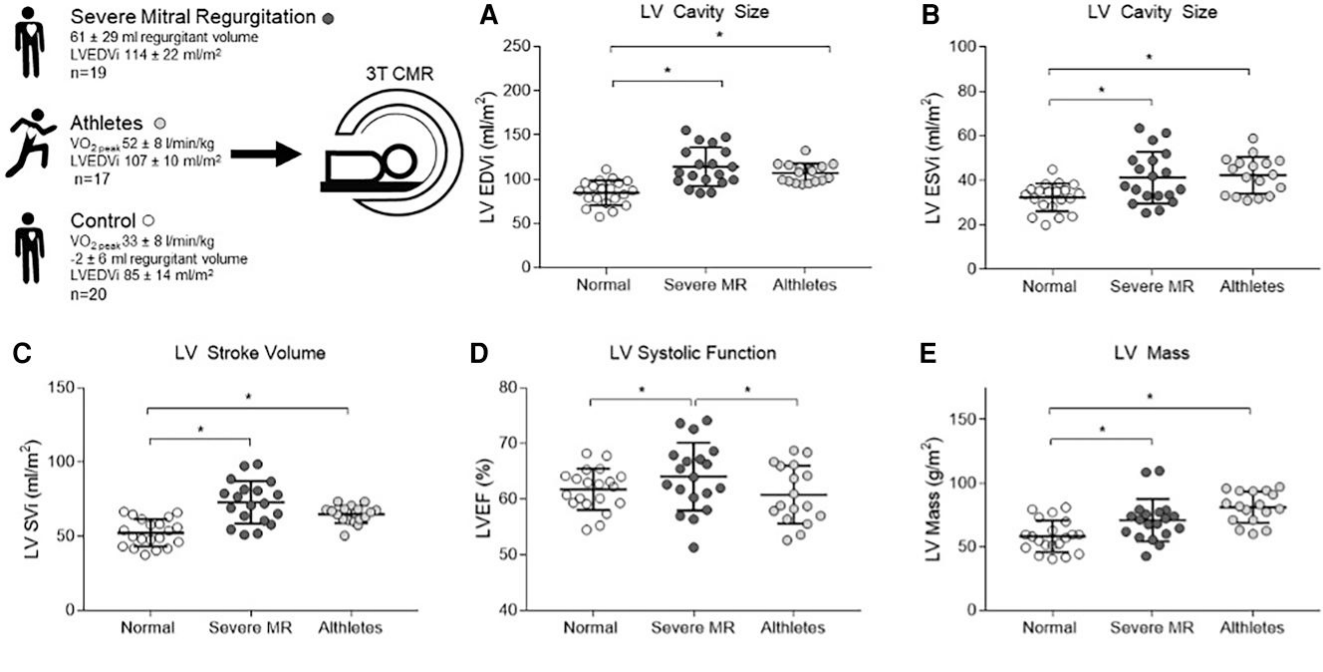
LV geometry in the study groups showing (*A*) LV end-diastolic volume (LVEDVi, mL/m^2^), (*B*) LV end-systolic volume (LVESVi, mL/m^2^), (*C*) LV stroke volume (LVSVi, mL/m^2^), (*D*) LV ejection fraction (LVEF, %), and (*E*) LV mass (g/m^2^), index to body surface area. Error bars shown are ±1SD. *P < 0.05 (one-way ANOVA Bonferroni corrected).

Mitral regurgitant volume was 61 ± 29 mL (regurgitant fraction 42 ± 13%; *Table 2*). Both severe MR and athletic training groups had dilated LV cavities (LVEDVi; MR 114 ± 22, athletes 107 ± 10 mL/m^2^) when compared to healthy volunteers (85 ± 14 mL/m^2^, *P* < 0.01, for both analyses), but LVEDV was not different between severe MR and athletes (*P* > 0.99). Stroke volume showed a similar pattern, being elevated in severe MR and athletic hearts when compared to healthy volunteers, but again not different between the two groups. As expected, athletes had higher LV mass than healthy volunteers (*P* = 0.03), but similar to severe MR (*P* = 0.29, *Figure 1*). LVEF was higher in severe MR than observed in the other two groups (*P* < 0.03), although it remained within the normal range. Right ventricular cavity size was also higher in athletes than in either the healthy volunteer or severe MR groups (*P* > 0.01 for both comparisons, *Table 2*). 3D circumferential (healthy volunteers −20.5 ± 2.2%, vs. MR −21.8 ± 3.3%, *P* = 0.15) and longitudinal strain (healthy volunteers −9.9 ± 2.4% vs. MR −8.4 ± 2.7%, *P* = 0.09) were not different.

**Table 2:** Baseline chamber dimensions and energetic measures

	Severe MR (*n* = 19)	Healthy volunteer (*n* = 20)	Athlete (*n* = 17)
**Mitral regurgitant volume (mL)**			
LV/RV stroke volume difference	61 (29)	−2 (6)*	−0.2 (7)^$^
LVSV—aortic forward flow	61 (27)	−6 (5)*	—
**Mitral regurgitant fraction (%)**			
LV/RV stroke volume difference/LVSV	42 (13)	−2 (7)*	−0.3 (6)^$^
(LVSV—aortic forward flow)/LVSV	42 (11)	−7 (5)*	—
**Left atrium**			
Maximum LA volume index (mL/m^2^)	73 (39)	32 (12)*	42 (11)^$^
LA ejection fraction (%)	48 (19)	61 (12)*	60 (8)^$^
**Left ventricle**			
LV end diastolic volume index (mL/m^2^)	114 (22)	85 (14)*	107 (10)^#^
LV end systolic volume index (mL/m^2^)	41 (12)	32 (6)*	41 (8)^#^
LV stroke volume index (mL/m^2^)	73 (14)	52 (9)*	66 (6)^#^
LV ejection fraction (%)	64 (6)	62 (4)*	62 (5)^$^
LV mass index (g/m^2^)	71 (17)	58 (12)*	77 (13)^#^
Total cardiac output (SV × HR, L/min)	10.4 (3.3)	6.0 (1.8)*	6.2 (1.3)^$^
Forward cardiac output (aortic flow × HR, L/min)	5.8 (1.7)	6.21 (1.8)	6.4 (1.3)
**Right ventricle**			
RV end diastolic volume index (mL/m^2^)	77 (24)	87 (16)	109 (11) ^#.$^
RV end systolic volume index (mL/m^2^)	35 (16)	33 (8)	45 (10)^#.$^
RV stroke volume index (mL/m^2^)	42 (11)	53 (8)*	64 (7)^#.$^
RV Ejection fraction (%)	56 (8)	62 (5)*	59 (6)
**Myocardial energetics**			
PCr/ATP ratio	1.56 (0.32)	2.08 (0.38)*	2.2 (0.28)^$^
Creatine kinase *k*_f_ adj (/s)	0.19 (0.10)	0.23 (0.11)	0.25 (0.16)
Creatine kinase flux (μmol/g/s)	1.8 (1.1)	2.8 (1.4)	3.1 (1.9)^$^

Parameters are reported as mean (SD). HR, heart rate; SV, stroke volume. Parameters differing significantly between severe MR and healthy volunteers are marked *P < 0.05. Parameters differing significantly between athletes and healthy volunteers are marked ^#^*P* < 0.05. Parameters differing significantly between athletes and severe MR are marked ^$^*P* < 0.05.

Overall, this confirmed the presence of severe MR and eccentric remodelling, and showed that LV cavity size and stroke volume were not different between the severe MR and athlete groups.

### Myocardial energetics

**Figure 2:**
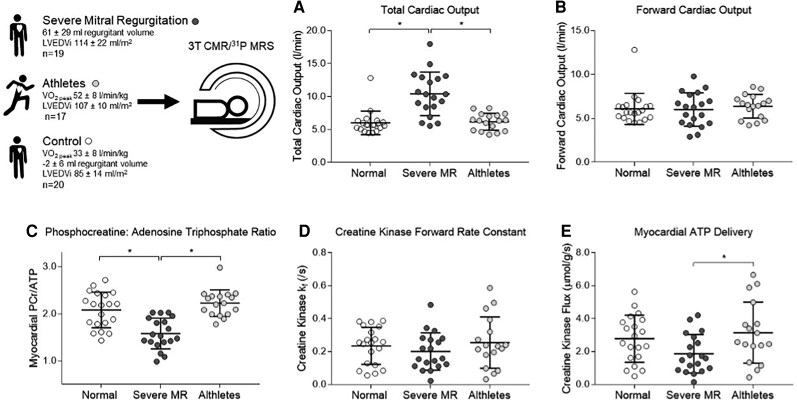
(*A*) total (LV stroke volume × heart rate), and (*B*) forward cardiac output (aortic forward flow × heart rate), (*C*) myocardial PCr/ATP, (*D*) creatine kinase forward rate constant, and (*E*) creatine kinase flux. Error bars are ± 1SD. *P < 0.05 (one-way ANOVA Bonferroni corrected).

**Figure 3:**
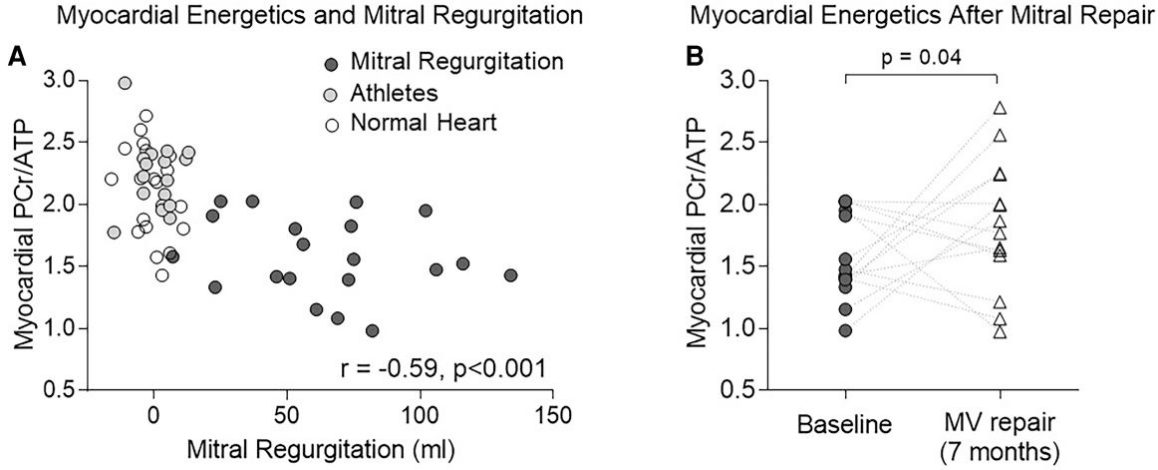
(*A*) Scatter plot of mitral regurgitant volume (LV-RV stroke volume) against PCr/ATP, (*B*) paired PCr/ATP values before and after MVR (*P* value 0.04, one-sided *t*-test, as pre-specified aim to investigate improvement in PCr/ATP with MVR).

Myocardial PCr/ATP ratio was significantly lower in severe MR (1.58 ± 0.32; *Table 2*) than in both healthy volunteers (2.08 ± 0.28) and athletes (2.23 ± 0.28, *P* < 0.001 in both analyses, *Figure 2*). In addition, PCr/ATP was related to the degree of MR (*r* −0.59, *P* < 0.001, *Figure 3*), LV stroke volume (*r* = −0.31, *P* = 0.054), and estimated stroke work (mean arterial pressure × SV, *r* −0.43, *P* = 0.007), but not to LVEF (*r* = −0.20, *P* = 0.23), whole heart 3D circumferential (*r* = 0.22, *P* = 0.18) or longitudinal strain (*r* = −0.03, *P* = 0.85).

Although in absolute terms the forward rate constant through CK (*k*_f_) was lowest in severe MR (0.20 ± 0.11/s) and highest in athletes (0.25 ± 0.16/s), it was not statistically different between the groups at rest (*P* = 0.46). CK flux (CK *k*_f_ × [PCr]) was lower in severe MR than in athletes (by 41%, Bonferroni corrected *P* = 0.039). On independent samples, Jonkckheere–Terpstra test for ordered medians (severe MR < healthy volunteers < athletes), there was an increasing median PCr/ATP (*P* = 0.02) and CK flux (*P* < 0.001) when comparing the groups in this order.

Overall, this shows that, when compared to athletic hearts with similar cavity dilatation, CK flux is reduced in severe MR, driven predominantly by a fall in phosphocreatine pool, which itself is related to mitral regurgitant severity and elevated stroke work.

### Creatine and total CK activity

HPLC of LV biopsies was used to determine whether the reduced PCr/ATP in severe MR was due to reduced total creatine pool. As LV biopsies from healthy volunteers were not available, these were compared with a group of non-volume-loaded hearts who were undergoing cardiac surgery as described above. This showed that total creatine levels were unlikely to be lower than in the normal heart (severe MR *n* = 7, 94.7 ± 26.2 vs. NonVol *n* = 6, 66.2 ± 14.5 nmol.mg^−1^ protein). CK total activity was measured in a smaller subset where biopsies were large enough. CK activity was numerically similar for the available *n* = 6 NonVol group (5.1 ± 1.27 IU/mg protein) and *n* = 2 severe MR (4.7 ± 1.72 IU/mg protein) samples. PCr/ATP, Ck *k*_f_, and CK flux were similar in the smaller biopsy groups to those observed in the larger study groups (see Supplementary data online, *Figure S1*).

Overall, this suggests that myocardial total creatine levels are unlikely to be decreased in severe MR and therefore the decrease in PCr/ATP is unlikely to be due to creatine pool depletion.

### Effect of increasing cardiac output in the normal heart

All 20 healthy volunteers underwent the dobutamine stress protocol. During dobutamine infusion, heart rate (60 ± 4 vs. 114 ± 4 beats per minute, *P* < 0.001) and total cardiac output (6.1 ± 1.8 vs. 11.6 ± 2.9 L/min, *P* < 0.001) increased. Although no change in PCr/ATP was observed during stress in the normal heart (PCr/ATP_rest_ 2.0 ± 0.6 vs. PCr/ATP_stress_ 1.9 ± 0.5, *P* = 0.28), median CK *k*_f_ increased 36% (*k*_f rest_ 0.25 (0.13, 0.35) vs. *k*_f stress_ 0.34 (0.25, 0.48), *P* < 0.001), and median CK flux increased 38% (CK flux_rest_ 2.9 (0.9, 4.4) vs. CK flux_stress_ 3.8 (2.2, 5.5) μmol/g/s, *P* = 0.011).

**Figure 4:**
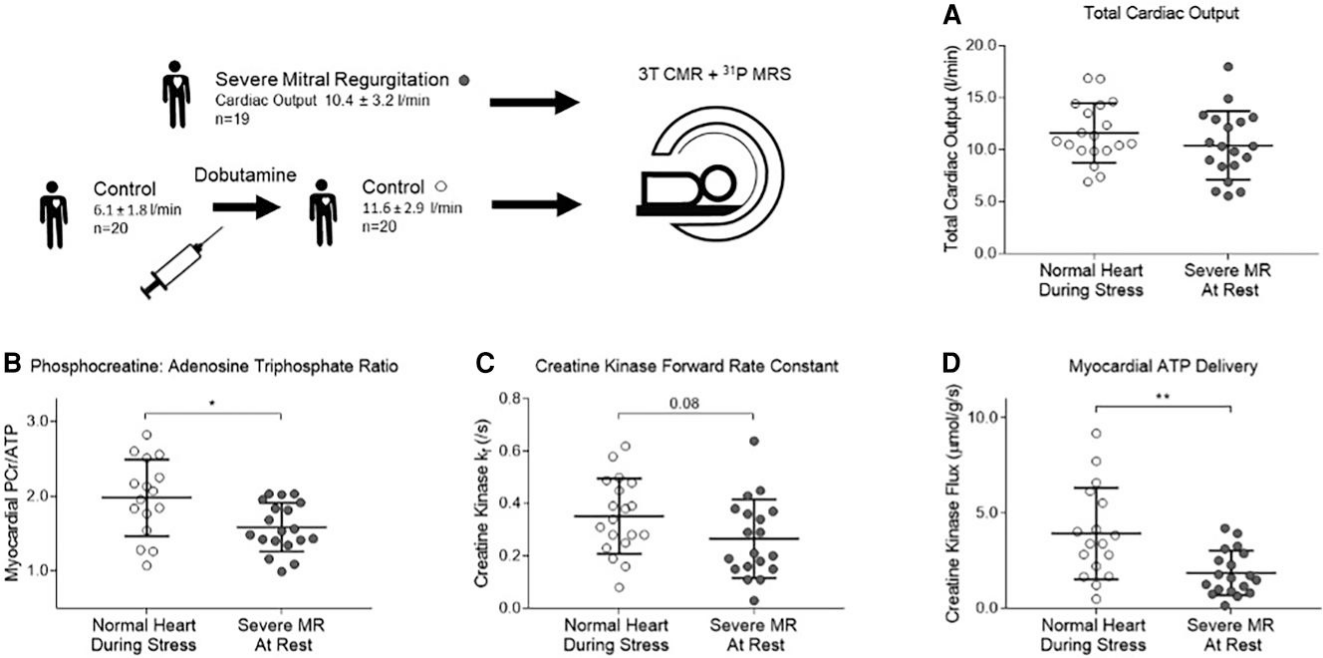
(*A*) Total cardiac output (stroke volume × heart rate), (*B*) PCr/ATP, (*C*) CK *k*_f_, and (*D*) CK flux in healthy volunteers during catecholamine stress and severe MR. Error bars are ±1SD, *P < 0.05, **P < 0.01.

Importantly, cardiac output achieved during dobutamine infusion was similar to that in the severe MR group at rest (healthy_stress_ 11.6 ± 2.9 L/min, vs. severe MR 10.4 ± 3.2 L/min, *P* = 0.24, *Figure 4*). Despite similar cardiac outputs, the severe MR group had lower mean PCr/ATP (by 26%, *P* < 0.05), median CK *k*_f_ (by 45%, *P* = 0.08), and CK flux (by 53%, *P* < 0.05) at rest than in the healthy heart during stress (*Figure 4*).

Overall, this shows that, when matched to the elevated cardiac output seen in severe MR, CK flux in normal hearts is over two-fold higher, as a result of both higher [PCr] and higher CK *k*_f_.

### The effect of mitral valve repair

Fourteen of 19 patients returned a median of 7.0 (6.2, 7.4) months after MVR (3 declined follow-up, 2 had permanent pacemaker insertion post-operatively). Alongside normalization of regurgitant volume (mean 0 ± 11mLs after MVR), LVMi decreased by 16%, LVEDVi by 36%, LVESVi by 27%, and LVSV by 42% (*P* < 0.001, *Table 3*). Mean arterial pressure was unchanged (*P* = 0.22), resulting in a reduced approximated LV stroke work (MAP × LVSV) by 42% (*Table 3*). LVEF reduced by 8% (relative) and 4.8% (absolute, *P* = 0.02). Myocardial energetics also improved post–operatively, with PCr/ATP increasing 17% from 1.55 ± 0.34 to 1.83 ± 0.53 (one-sided *P* = 0.040, *Figure 3B*).

**Table 3:** Anthropometrics, chamber dimensions, and energetics after MVR

	Pre-op (*n* = 14)	Late follow-up (*n* = 14)	*P*-value
**Anthropometric**			
Body surface area (m^2^)	1.89 (0.26)	1.91 (0.26)	0.13
Mean arterial pressure (MAP, mmHg)	94 (9)	91 (8)	0.22
**Mitral regurgitant volume (mL)**	59 (30)	0 (11)	<0.001
LV/RV stroke volume difference	60 (29)	−3 (9)	<0.001
LVSV—aortic forward flow			
**Exercise capacity**			
6MWT (m)	531 (110)	537 (112)	0.59
**Left ventricle**			
LV end diastolic volume index (mL/m^2^)	111 (22)	71 (13)	<0.001
LV end systolic volume index (mL/m^2^)	40 (11)	30 (9)	<0.001
LV stroke volume index (mL/m^2^)	71 (14)	42 (7)	<0.001
LV ejection fraction (%)	64 (6)	59 (8)	0.02
LV mass index (g/m^2^)	66 (11)	55 (10)	<0.001
LV mass: volume (g/mL)	0.59 (0.56–0.67)	0.78 (0.71–0.81)	<0.001
Stroke work (MAP × SV)	12 484 (3058)	7226 (1643)	<0.001
**Right ventricle**			
RV end diastolic volume index (mL/m^2^)	72 (60–85)	69 (58–74)	0.63
RV end systolic volume index (mL/m^2^)	30 (23–39)	27 (25–29)	0.04
RV stroke volume index (mL/m^2^)	40 (6)	42 (10)	0.40
RV ejection fraction (%)	55 (9)	59 (6)	0.04
**Myocardial energetics**			
PCr/ATP ratio	1.55 (0.34)	1.83 (0.53)	0.04

*P*-value for PCr/ATP is one-sided; others are two-sided.

## Discussion

Risk stratification and timing of valve intervention in severe MR remain difficult, and newer risk markers are needed. We have shown, despite apparently normal LV systolic function, that severe MR when matched for stroke volume (against the normal heart during stress) and eccentric remodelling (against the athletic heart) is associated with reduced PCr/ATP, CK *k*_f_, and CK flux. In addition, we have shown this PCr/ATP reduction is related to regurgitant severity, predates LVEF decline, is not related to creatine pool depletion, and resolves with successful valve repair. This suggests myocardial energetics could be a valuable risk marker in MR.

### Myocardial energetic response to mitral regurgitation

Whilst confirming the reduced PCr/ATP previously reported in MR,^7^ we have shown for the first time that CK flux is also reduced. In addition, we show that the total creatine pool is also unlikely to be decreased. This combination would result in elevated free creatine (free Cr) and suggests that either mitochondrial ATP production or mito-CK activity is unable to maintain the fraction of the total creatine pool that is phosphorylated.

Although creatine is a stimulant for oxidative phosphorylation,^15^ when free creatine rises, intracellular free adenosine diphosphate (ADP) also increases (being determined as [ADP] = ([ATP].[free Cr])/([PCr][H^+^]CK Keq)).^16^ It seems likely that the energy buffering role of CK, which serves to keep free [ADP] levels low and the free energy change of ATP hydrolysis (Δ*G*_ATP_) high, at the sites of ATP hydrolysis, is impaired in severe MR, in a similar fashion to that seen in cardiac creatine over-expression models,^14^ where hypertrophy and heart failure develop with supra-normal creatine levels.

As the Creatine pool is unlikely to be lower, the cause of the fall in PCr/ATP observed in severe MR in this study is likely to be a mismatch between elevated demand and oxidative phosphorylation capacity. Altered substrate selection is one possible explanation underpinning this. Experimental data have shown that fatty acid metabolism is reduced in the chronic volume-loaded heart from aorto-caval fistula.^17^ As such, if mitochondrial ATP production falls below the increased demand imposed by the increased stroke work of MR, reversible PCr depletion would occur.

### Myocardial energetic response in athletes

In comparison, despite a similar pattern of eccentric hypertrophy, PCr/ATP, CK, and CK flux were not reduced in athletic hearts. This suggests that, in athletic cardiac remodelling, oxidative phosphorylation and CK activity are able to keep up with the increased metabolic demand imposed by volume loading. As PCr/ATP was not reduced in athletic heart, suggesting that when CK activity (flux) is maintained, this prevents the increased free creatine pool and subsequent reduction in Δ*G*_ATP_ may be avoided.

### Effect of acutely increasing cardiac output

In this study, we have shown that the normal heart can increase myocardial ATP delivery by increasing CK flux (by 36%) when cardiac output is increased to that seen in severe MR. This has been observed previously in rodents,^18^ and non-obese humans.^8^ This would suggest that the observed CK *k*_f_ in severe MR is only 59% of what would be expected for the level of cardiac output.

When the reduced PCr pool (by 26%) is also taken into account, ATP delivery through CK is only 47% of what would be expected. When coupled with the effects of elevated free creatine described above, this represents a very significant reduction in myocardial energetic reserve, and if further assuming unchanged [Pi] and [ADP], the Δ*G* ∼ ATP would be significantly lower.

### Effect of mitral valve repair

We have shown that with successful MVR, there is a significant increase in PCr/ATP, suggesting that the energetic impairment is at least partially reversible. The associated 16% post-operative fall in LV mass indicates significant reverse remodelling, and with it, a reduced energetic demand would be expected. As creatine pool was not lower pre-operatively, the increase in PCr/ATP accompanying this reverse remodelling also suggests a re-balancing of ATP demand and supply accompanies this reduction in volume loading. This would be in keeping with the observation using ^11^C-acetate positron emission tomography, that MVR can improve stroke volume without increasing oxidative metabolism, and results in improved forward myocardial efficiency.^19^

## Limitations

These findings should be set in the context of the disease stage studied. This severe MR cohort did not undergo ‘early’ repair as many had class 1 indications: 16 of 19 admitted dyspnoea (although exercise testing to prove asymptomatic status was not performed), 10–12 had regurgitant volume ≥50 mL. Eleven had regurgitant fraction >40%, and 7 had LVESD ≥ 40 mm; RVEF was reduced; mean 6 min walk distances were lower and 4 had atrial fibrillation mean. Pulmonary artery pressures were not assessed, which limits comprehensive haemodynamic characterization, particularly with respect to right ventricular adaptation.

Groups were matched for forward cardiac output, but without invasive studies, it cannot be certain whether this translates to equivalent expected rates of myocardial oxygen consumption per gram myocardium, of mean wall stress, or of normalized stroke work (area within a pressure-volume loop), and thus to equivalent expected CK fluxes. To our knowledge, such studies have not been performed, although it is known that chronic decompensated MR is associated with an increase in normalized stroke work.^3^

The CK system in severe MR was not interrogated during further increased myocardial work, when the CK system is thought to be most important. However, as contractile reserve testing is contraindicated in symptomatic severe MR, this was not possible. Additionally, in the subset of patients who underwent biopsy for creatine analysis, the non-volume-loaded surgical comparator group included individuals with mitral stenosis and aortic aneurysm. While these patients did not have LV volume overload, latent myocardial energetic alterations cannot be excluded and may have influenced myocardial creatine levels.

Repeat *k*_f_ assessment was not performed at the follow-up time point. However, as *k*_f_ at rest in severe MR was not different from healthy volunteers at rest, this would not be expected to have changed at follow-up.

Groups were not well age-matched to the severe MR group, which may have magnified the differences in pre-operative PCr/ATP values. Moreover, sample sizes precluded adjustment for other factors that may impact PCr/ATP, such as blood pressure and medications. However, the post-operative rise in PCr/ATP argues that volume loading in severe MR was a significant factor contributing to the lower PCr/ATP.

## Conclusion

Despite a common volume-loading stimulus, physiological remodelling from athletic training and pathological remodelling from MR are prognostically different. Here we have shown that, when compared to athletes and normal hearts under catecholamine stress, the elevated cardiac output state in severe MR is associated with reduced PCr/ATP, CK *k*_f_, and CK flux. When coupled with a constant creatine pool, these changes will have marked negative effects on the free energy of hydrolysis of ATP, required for contractile function. This suggests if severe enough, this may dispose to transition to failure, or failure to improve post-intervention. Importantly, with timely intervention, these energetic impairments appear to be at least partially reversible. Whether non-invasive metabolic imaging techniques could be used to identify those at risk of transition to failure, or predict recovery post–MVR, is now worthy of further investigation.

## Supplementary Material

qyaf146_Supplementary_Data

## Data Availability

Data will be made available upon reasonable request to the corresponding author.

## Lead author biography

Dr Peterzan achieved his BSc and MBBS from Imperial College London in 2007 and 2009, respectively. He obtained his DPhil in Medical Sciences from St Catherine's College, University of Oxford, in 2020 with a focus on creatine kinase, the phosphotransfer part of cardiac metabolism, in acquired human myocardial hypertrophy. After completing cardiology training on the South London rotation in 2022, he completed a post-CCT fellowship in advanced heart failure at Harefield Hospital before taking up a position as consultant cardiologist with interest in cardio-metabolic medicine at St George's Hospital in late 2023.
