# Supplementary material for: Cardiac energetics in severe mitral regurgitation: relationship with eccentric hypertrophy, stroke volume, and effects of valve repair
Source: Eur Heart J Imaging Methods Pract. 2025 Nov 25;3(4):qyaf146. doi: 10.1093/ehjimp/qyaf146 (PMC12700164; doi:10.1093/ehjimp/qyaf146)
Supplement: qyaf146_Supplementary_Data [file qyaf146_supplementary_data.docx]

## Expanded Methods

## Left ventricular imaging

A short stack of cine images was obtained using a 24 channel spine matrix, 6 channel body matrix and steady-state free precession sequences. In brief, all images were ECG-gated and taken during end-expiratory breath-hold. Typical SSFP sequence parameters were slice thickness 8 mm, gap 2 mm, retrospective gating, TE 1.5 ms, TR 46 ms, flip-angle 50º, FOV 400 mm, matrix size 256 in frequency encode direction. Prospective gating was used if the participant was in atrial fibrillation. LV endocardial and epicardial contours were performed manually. LV ventricular indices was normalised to body surface area (BSA) using the Mosteller formula. Late gadolinium imaging was performed to exclude myocardial infarction according to standard clinical protocol. 3D Left ventricular circumferential and longitudinal strain measures were derived from manually contoured short axis and long axis images using the feature tracking module within cvi42.

# Cardiac ^31^P-Magnetic Resonance Spectroscopy

### Phosphocreatine: Adenosine Triphosphate Ratio (PCr/ATP)

Participants were positioned prone over a three-element dual-tuned ^1^H/^31^P surface coil at magnet isocentre. An ~10 min non-gated 3D acquisition-weighted ultra-short echo time CSI sequence was run as previously described. Parameters included: acquisition matrix size 16 × 16 × 8 voxels, field of view 240 × 240 × 200 mm^3^, nominal voxel size 5.6 ml, 10 averages at the centre of k-space, fixed TR per-subject (910-1010 ms depending on specific absorption rate constraints), centre frequency 250 Hz from PCr. The PCr/ATP ratio reported is the blood- and saturation-corrected PCr/average ATP ratio, averaged over the two most basal septal voxels. Spectral analyses were performed using “OXSA”, an open-source MATLAB implementation of the AMARES algorithm.

### Creatine Kinase Pseudo First Order Forward Rate Constant (CK k_f_)

Resting CK k_f_ was estimated using triple repetition time saturation transfer (TRiST) adapted for use with a transmit/receive 10 cm loop radiofrequency coil (PulseTeq Ltd, Chobham, UK) as previously described (Peterzan et al., 2020; Rayner et al., 2020). In brief, participants were scanned supine; ^1^H localisers confirmed coil position; then a 1D phase-encoded CSI matrix (16 slices, 160 mm) was used to acquire four sets of ^31^P spectra. These were: a fully-relaxed acquisition (TR 15 s, 2 averages, 9 min), two acquisitions with selective γATP saturation (TR 1.5 then 9.5 s, 18 then 8 averages, 11 then 21 min), and one with control saturation mirrored around PCr (TR 15 s, 2 averages, 9 min). Spectral analysis was performed using custom software(Purvis et al., 2017). The pseudo-first order forward rate constant of CK was then calculated according to:

$$k_{\text{f}}^{\text{CK}}=\frac{1}{T_{1}^{*}}\left(\frac{M_{\text{PCr}}^{\text{Ctrl}}}{M'_{\text{PCr}}}-1\right).$$

Forward CK flux was calculated by CK k_f_ × [PCr], where [PCr] is estimated by multiplying PCr/ATP by literature values for [ATP] (5.7 μmol/g wt weight). We multiplied all k_f_ values by a previously validated factor of 1.333 to adjust for supine scanning (Clarke et al., 2019).

### Energetic measurements during dobutamine stress

Following baseline k_f_ assessment, two further sets of ^31^P spectra were acquired upon achievement of target heart rate, one with control saturation mirrored around PCr (TR 15 s, 2 averages, 9 min) and one with selective γATP saturation (TR 15 s, 2 averages, 9 min), as previously described (Clarke et al., 2019).

# Cardiopulmonary exercise testing (CPET)

All participants underwent bicycle CPET with breath-by-breath respiratory gas measurement and individualised protocols. Typical protocols in healthy volunteers aimed to achieve peak VO_2_ at 10-12 minutes and comprised: rest 2 min, unloaded 2 min, starting work rate 25 W, continuous ramp increase of 15-20 W/min, recovery 3 min. Athletes underwent longer protocols, starting at 100 W and with stepped increases of 25 W every 3 min. Criteria for protocol completion were plateauing of VO2 and one of: achievement of maximum heart rate, respiratory exchange ratio >1.1, or inability to maintain further work increases. (No safety criteria for stopping a test were met). Minute oxygen consumption (VO_2_), heart rate, and anaerobic threshold as a percentage of peak VO_2_ are presented. One author (DD) blinded to group assignment read the AT as the first inflection point of the VCO_2_/VO_2_ slope.

1. **
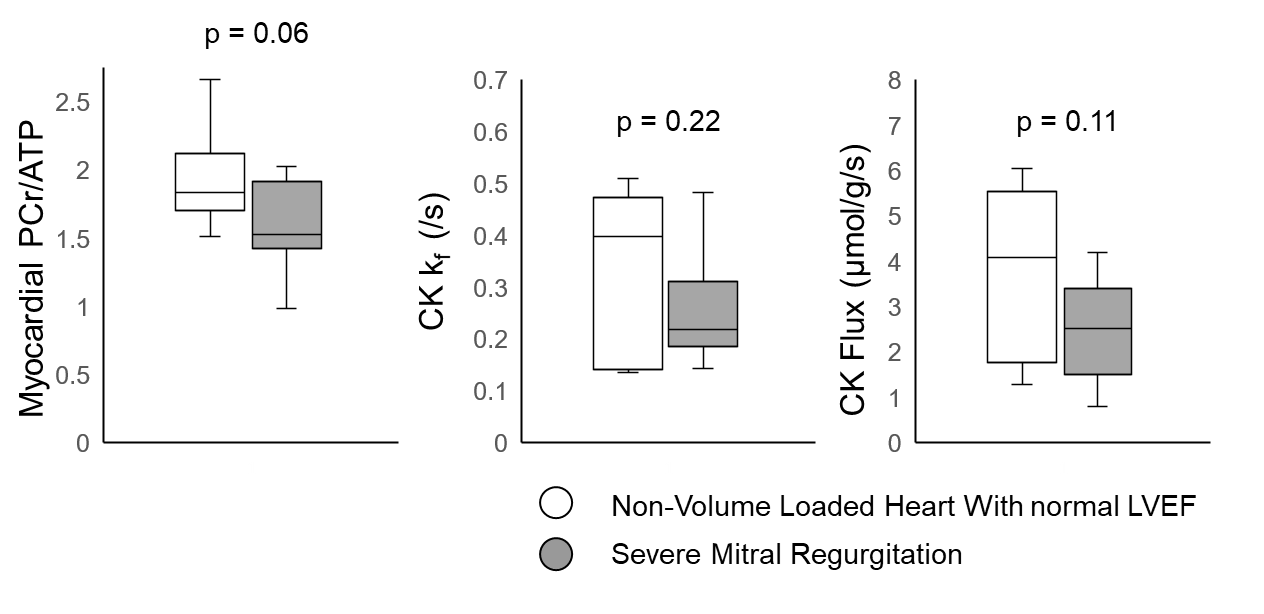
Supplementary Figure**

**Supplemental Figure 1.** PCr/ATP, k_f_ and CK resuts for only those patients who consented and donated LV biopsy during surgery.

Clarke, W. T., Peterzan, M. A., Rayner, J. J., Sayeed, R. A., Petrou, M., Krasopoulos, G., Lake, H. A., Raman, B., Watson, W. D., Cox, P., Hundertmark, M. J., Apps, A. P., Lygate, C. A., Neubauer, S., Rider, O. J., & Rodgers, C. T. (2019). Localized rest and stress human cardiac creatine kinase reaction kinetics at 3 T. *NMR Biomed*, *32*(6), e4085. <https://doi.org/10.1002/nbm.4085>

Peterzan, M. A., Clarke, W. T., Lygate, C. A., Lake, H. A., Lau, J. Y. C., Miller, J. J., Johnson, E., Rayner, J. J., Hundertmark, M. J., Sayeed, R., Petrou, M., Krasopoulos, G., Srivastava, V., Neubauer, S., Rodgers, C., & Rider, O. J. (2020). Cardiac Energetics in Patients with Aortic Stenosis and Preserved versus Reduced Ejection Fraction. *Circulation*. <https://doi.org/10.1161/CIRCULATIONAHA.119.043450>

Purvis, L. A. B., Clarke, W. T., Biasiolli, L., Valkovic, L., Robson, M. D., & Rodgers, C. T. (2017). OXSA: An open-source magnetic resonance spectroscopy analysis toolbox in MATLAB. *PLoS One*, *12*(9), e0185356. <https://doi.org/10.1371/journal.pone.0185356>

Rayner, J. J., Peterzan, M. A., Watson, W. D., Clarke, W. T., Neubauer, S., Rodgers, C. T., & Rider, O. J. (2020). Myocardial Energetics in Obesity: Enhanced ATP Delivery Through Creatine Kinase with Blunted Stress Response. *Circulation*. <https://doi.org/10.1161/CIRCULATIONAHA.119.042770>
